# Supplementary figures and images for: Ciprofloxacin Concentrations 1/1000th the MIC Can Select for Antimicrobial Resistance in N. gonorrhoeae—Important Implications for Maximum Residue Limits in Food
Source: Antibiotics (Basel). 2022 Oct 18;11(10):1430. doi: 10.3390/antibiotics11101430 (PMC9598464; doi:10.3390/antibiotics11101430)

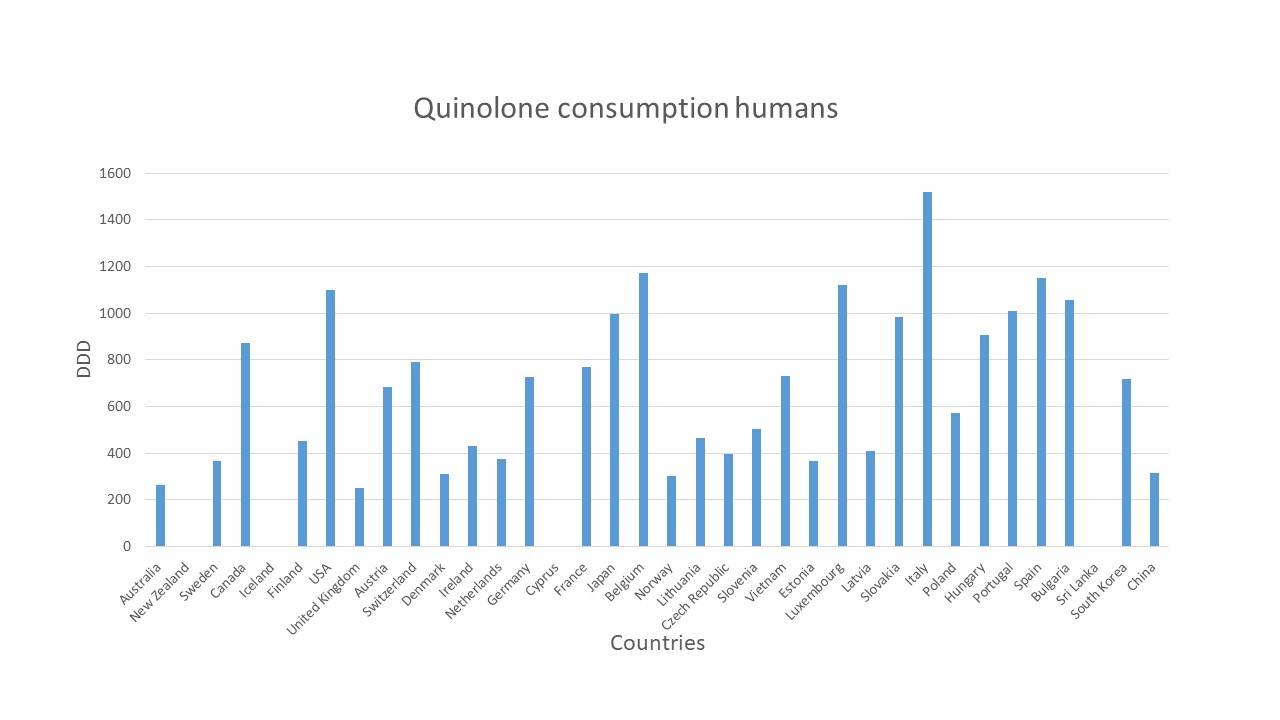

Supplement: Supplementary file 1 [file antibiotics-11-01430-s001.zip › antibiotics-1943801-supplementary-final/Supplementary 1.JPG]

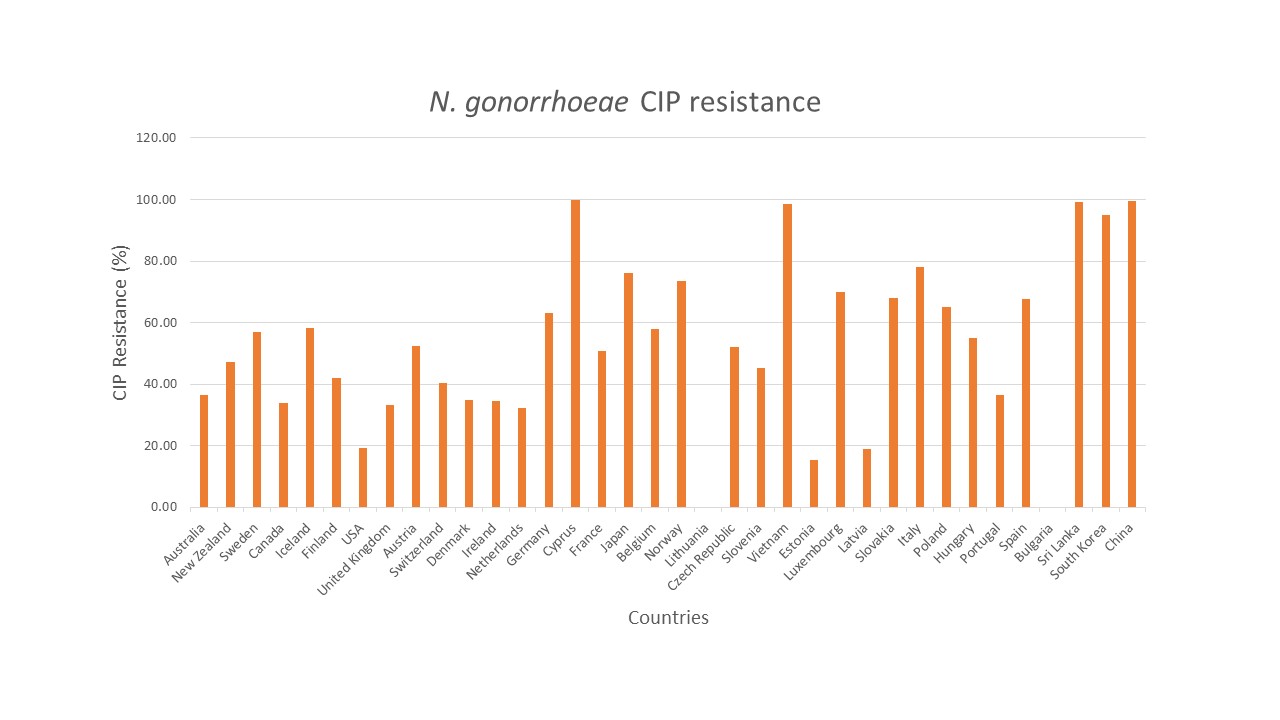

Supplement: Supplementary file 1 [file antibiotics-11-01430-s001.zip › antibiotics-1943801-supplementary-final/Supplementary 2.JPG]

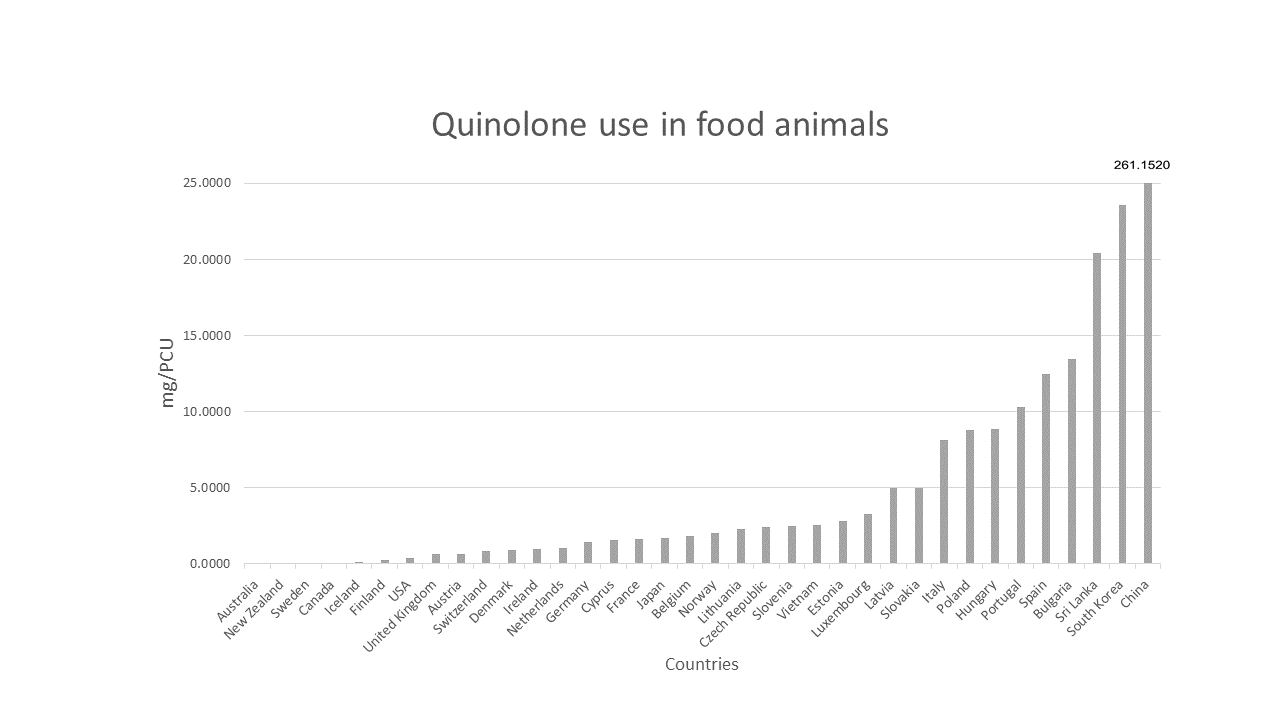

Supplement: Supplementary file 1 [file antibiotics-11-01430-s001.zip › antibiotics-1943801-supplementary-final/Supplementary 3.PNG]
